# A New Family of Continuous Probability Distributions

**DOI:** 10.3390/e23020194

**Published:** 2021-02-05

**Authors:** M. El-Morshedy, Fahad Sameer Alshammari, Yasser S. Hamed, Mohammed S. Eliwa, Haitham M. Yousof

**Affiliations:** 1 Department of Mathematics, College of Science and Humanities in Al-Kharj, Prince Sattam bin Abdulaziz University, Al-Kharj 11942, Saudi Arabia; f.alshammari@psau.edu.sa; 2 Department of Mathematics, Faculty of Science, Mansoura University, Mansoura 35516, Egypt; mseliwa@mans.edu.eg; 3 Department of Mathematics and Statistics, College of Science, Taif University, Taif 21944, Saudi Arabia; yasersalah@tu.edu.sa; 4 Department of Statistics, Mathematics and Insurance, Benha University, Benha 13518, Egypt; haitham.yousof@fcom.bu.edu.eg

**Keywords:** poisson distribution, generalized exponential distribution, compounding, Farlie-Gumbel-Morgenstern, clayton copula, Ali-Mikhail-Haq copula, modeling, Lomax distribution, kernel density estimation

## Abstract

In this paper, a new parametric compound G family of continuous probability distributions called the Poisson generalized exponential G (PGEG) family is derived and studied. Relevant mathematical properties are derived. Some new bivariate G families using the theorems of “Farlie-Gumbel-Morgenstern copula”, “the modified Farlie-Gumbel-Morgenstern copula”, “the Clayton copula”, and “the Renyi’s entropy copula” are presented. Many special members are derived, and a special attention is devoted to the exponential and the one parameter Pareto type II model. The maximum likelihood method is used to estimate the model parameters. A graphical simulation is performed to assess the finite sample behavior of the estimators of the maximum likelihood method. Two real-life data applications are proposed to illustrate the importance of the new family.

## 1. Introduction and Genesis

In statistical literature, we always assume that every real phenomenon can be modeled by some lifetime distributions. If we know this distribution(s), we can then analyze our phenomenon, as many lifetime distributions have been developed in this regard. The well-known Poisson distribution is one of the famous distributions that was also defined and studied as a new family of continuous distribution in the concept of compounding. Using the Poisson G family, several compound lifetime G families have been proposed and studied. In the compounding method, there are two different approaches available; one is by using zero truncated power series (ZTPS) distribution and the other by using zero truncated Poisson (ZTP) distribution directly with other continuous distributions. A comprehensive survey regarding the Poisson G models was recently proposed by [1].

In this paper, we propose and study a new family of distributions using ZTP distribution with a strong physical motivation. Suppose that a system has N (a discrete random variable) subsystems functioning independently at a given time, where N has a ZTP distribution with parameter λ and the failure time of *i*th component $Y_{i}|i=1,2,\ldots$ (say) is independent of N. It is the conditional probability distribution of a Poisson-distributed random variable (RV), given that the value of the RV is not zero. The probability mass function (PMF) of N is given by

$$P_{\lambda }\left(N=n\right)=\frac{\lambda^{n}exp(-\lambda )}{\Gamma \left(1+n\right){𝓒}_{\lambda }}{|}_{\left(n=N\right)\;and\;{𝓒}_{\lambda }=1-exp\left(-\lambda \right)}.$$

Note that for ZTP RV, the expected value E(N|λ) and variance V(N|λ) are, respectively, given by $E(N|\lambda )=\lambda /{𝓒}_{\lambda }$ and $V(N|\lambda )=\frac{\lambda \left(1+\lambda \right)}{{𝓒}_{\lambda }}-\frac{\lambda^{2}}{{𝓒}_{\lambda }^{2}}.$ Suppose that for each subsystem, the failure time (i.e., *i*th component) has the generalized exponential generator (GE-G) defined by the cumulative distribution function (CDF)

(1) $${𝓗}_{\theta ,\beta ,\underline{\xi }}\left(x\right)={\left[1-{Ϛ}_{\beta ,\underline{\xi }}\left(x\right)\right]}^{\theta },\theta ,\beta >0\;and\;x\in R,$$

where the function ${Ϛ}_{\beta ,\underline{\xi }}\left(x\right)=exp\left[-\beta \Delta_{\underline{\xi }}\left(x\right)\right]$, $\Delta_{\underline{\xi }}\left(x\right)=G_{\underline{\xi }}\left(x\right)/{\bar{G}}_{\underline{\xi }}\left(x\right)$ refers to the odd ratio function (ORF), $G_{\underline{\xi }}\left(x\right)$ refers to the base-line CDF with parameters vector $\underline{\xi }$, ${\bar{G}}_{\underline{\xi }}\left(x\right)=1-G_{\underline{\xi }}\left(x\right)$ refers to the base-line survival function (SF) of the base-line model and β>0 is a shape parameter, $G_{\underline{\xi }}\left(x\right)$ is the CDF of the base-line model, and $\frac{d}{dx}G_{\underline{\xi }}\left(x\right)=g_{\underline{\xi }}\left(x\right)$ is the probability density function (PDF) of the base-line model. Staying in (1) and for β=1, the GE-G reduces to exponential G (E-G) (see [2]). Let $Y_{i}$ denote the failure time of the *i*th subsystem, and let

$$X=min\left\{Y_{1},Y_{2},\cdots ,Y_{N}\right\},$$

Then, the conditional CDF of *X* given *N* is

$$F\left(x|N\right)=1-\mathrm{Pr}\left(X>x|N\right)=1-{\left[1-{𝓗}_{\theta ,\beta ,\underline{\xi }}\left(x\right)\right]}^{N}=1-{\left\{1-{\left[1-{Ϛ}_{\beta ,\underline{\xi }}\left(x\right)\right]}^{\theta }\right\}}^{N}.$$

Therefore, the unconditional CDF of *X*, as described in [3,4,5,6,7,8,9], can be expressed as

(2) $$F_{\underline{V}}\left(x\right)={𝓒}_{\lambda }^{-1}\left(1-exp\left\{-\lambda {\left[1-{Ϛ}_{\beta ,\underline{\xi }}\left(x\right)\right]}^{\theta }\right\}\right),x\in R$$

The CDF in (2) is called the Poisson generalized exponential G (PGEG) family, **V** = $\left(\lambda ,\theta ,\beta ,\underline{\xi }\right)$ is the parameter vector of the PGE-G family. The corresponding PDF can be derived as

(3) $$f\left(x\right)=\lambda {𝓒}_{\lambda }^{-1}h_{\theta ,\beta ,\underline{\xi }}\left(x\right)exp\left\{-\lambda {\left[1-{Ϛ}_{\beta ,\underline{\xi }}\left(x\right)\right]}^{\theta }\right\},x,\lambda >0,$$

where the function $h_{\theta ,\beta ,\underline{\xi }}\left(x\right)=d{𝓗}_{\theta ,\beta ,\underline{\xi }}\left(x\right)/dx$. Or, the PDF due to (3) can be re-expressed as

(4) $$f_{\underline{V}}\left(x\right)=\lambda \beta \theta {𝓒}_{\lambda }^{-1}\frac{g_{\underline{\xi }}\left(x\right){Ϛ}_{\beta ,\underline{\xi }}\left(x\right)}{{\bar{G}}_{\underline{\xi }}{\left(x\right)}^{2}{\left[1-{Ϛ}_{\beta ,\underline{\xi }}\left(x\right)\right]}^{1-\theta }}\underset{A_{\lambda ,\theta ,\beta }\left(x\right)}{\underbrace{exp\left\{-\lambda {\left[1-{Ϛ}_{\beta ,\underline{\xi }}\left(x\right)\right]}^{\theta }\right\}}},x\in R.$$

A RV X having PDF (4) is denoted by X∼ PGE-G $\left(\underline{V}\right)$. Some special cases of the PGE-G family are listed in Table 1.

**Figure 1:**
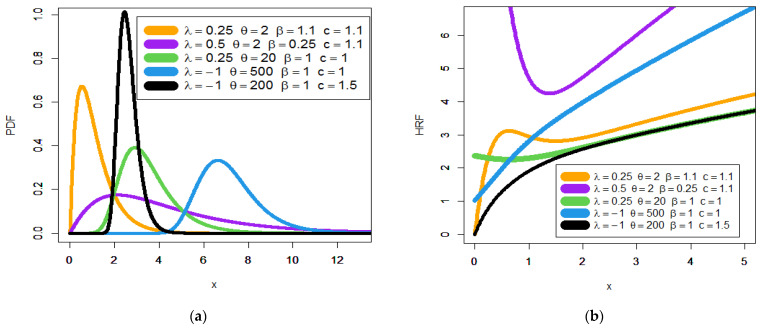
Plots of the PGEPII probability density function (PDF) (**a**) and PGEPII hazard rate function (HRF) (**b**).

**Figure 2:**
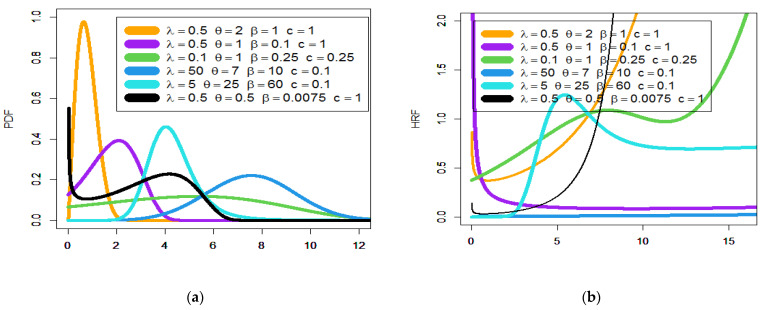
Plots of the PGEE PDF (**a**) and PGEE HRF (**b**).

Note that Γ(.) refers to the gamma function and γ(.,.) refers to the incomplete gamma function. Figure 1 gives some plots of the Poisson generalized exponential-Pareto type II (PGEPII) PDF (a) and PGEPII hazard rate function (HRF) (b) for some carefully selected parameters value. Figure 2 presents some plots of the Poisson generalized exponential-exponential (PGEE) PDF (a) and PGEE HRF (b) for some carefully selected parameters value. Based on Figure 1a, it is noted that the PDF of the PGEPII can be “asymmetric right-skewed function” and “symmetric” with different shapes. Based on Figure 2a, it is seen that the PDF of the PGEE can be “asymmetric right-skewed function”, “asymmetric left-skewed function”, “bimodal”, and “symmetric” with different shapes. Based on Figure 1b, it is noted that the HRF of the PGEPII can be “upside down bathtub (λ=4,θ=2,β=1,c=1.55)”, “bathtub (λ=6,θ=1,β=1,c=1.75)”, “decreasing-constant (λ=θ=β=c=1)”, “increasing-constant (λ=−1,θ=β=c=1)”, and “increasing (λ=−1,θ=2,β=1,c=1.5)”. Based on Figure 2b, it is noted that the HRF of the PGEE can be “bathtub (λ=0.5,θ=0.5,β=0.75,c=0.25)”, “decreasing-constant (λ=5,θ=0.25,β=c=0.1)”, “upside down bathtub (λ=10,θ=1,β=c=0.25)”, “constant (λ=10,θ=2,β=c=0.05)”, and “increasing (**J**-shape) (λ=0.5,θ=0.5,β=0.0007,c=1)”. 

The new family could be useful in modeling

1-The real datasets with “asymmetric monotonically increasing HRF”, as illustrated in Section 6.

2-The real datasets that have no extreme values, as shown in Section 6.

3-The real datasets whose nonparametric Kernel density is symmetric, as given in Section 6 (Figure 11).

The PGE-G family proved its superiority against many well-known families as shown below:

1-In modeling the failure times of the aircraft windshield, the PGE-G family is better than the special generalized mixture G family, the odd log-logistic G family, the Burr-Hatke G family, the transmuted Topp-Leone G family, the Gamma G family, the Kumaraswamy G family, the McDonald G family, the exponentiated G family, and the proportional reversed hazard rate G family under the Akaike information criteria, consistent information criteria, Bayesian information criteria, and Hannan–Quinn information criteria. 

2-In modeling the service times of the aircraft windshield, the PGE-G family is better than the special generalized mixture G family, the odd log-logistic G family, the Burr-Hatke G family, the transmuted Topp-Leone G family, the Gamma G family, the Kumaraswamy G family, the McDonald G family, the exponentiated G family, and the proportional reversed hazard rate G family under the Akaike information criteria, consistent information criteria, Bayesian information criteria, and Hannan-Quinn information criteria. 

## 2. Copula

For facilitating the mathematical modeling of the bivariate RVs, we derived some new bivariate PGE-G (Bv-PGE-G)-type systems of distributions using “Farlie-Gumbel-Morgenstern copula” (FGMCp for short) copula ([10,11,12,13,14,15]), modified FGMCp (see [16] for details) that contains for internal types, ” Clayton copula (CCp)” (see [17] for details), “Renyi’s entropy copula (RECp)” [18], and Ali-Mikhail-Haq copula [19]. The multivariate PGE-G (Mv PGE-G) type can be easily derived based on the Clayton copula. However, future works may be allocated to study these new models. 

### 2.1. BvPGE-G Type via CCp

Let us assume that $X_{1}∼$ PGE-G$\left({\underline{V}}_{1}\right)$ and $X_{2}∼$ PGE-G$\left({\underline{V}}_{2}\right)$. The CCp depending on the continuous marginal functions $\bar{w}=1-w$ and $\bar{\varpi }=1-\varpi$ can be considered as

(5) $$C_{\Omega }\left(\bar{w},\bar{\varpi }\right)={\left[\underset{}{\max}\left({\bar{w}}^{-\Omega }+{\bar{\varpi }}^{-\Omega }-1\right);0\right]}^{-\frac{1}{\Omega }},\Omega \in \left[-1,\infty \right)-\left\{0\right\},\bar{w}\in \left(0,1\right)\;and\;\bar{\varpi }\in \left(0,1\right)$$

Let $\bar{w}=1-F_{{\underline{V}}_{1}}\left(x_{1}\right){|}_{{\underline{V}}_{1}}$, $\bar{\varpi }=1-F_{{\underline{V}}_{2}}\left(x_{2}\right){|}_{{\underline{V}}_{2}}$ and

$$F_{{\underline{V}}_{i}}\left(x_{i}\right){|}_{i=1,2}={𝓒}_{\lambda_{i}}^{-1}\left(1-exp\left\{-\lambda_{i}{\left[1-{Ϛ}_{\beta_{i},{\underline{\xi }}_{i}}\left(x_{i}\right)\right]}^{\theta_{i}}\right\}\right).$$

Then, the BvPGE-G-type distribution can be obtained from (5). A straightforward multivariate PGE-G (m-dimensional extension) via CCp can be easily derived analogously. The m-dimensional extension via CCp is a function operating in ${\left[0,1\right]}^{m}$, and in that case, $x_{i}$ is not a value in [0,1] necessarily.

### 2.2. BvPGE-G Type via RECp

Following [18], the RECp can be derived as $C\left(w,\varpi \right)=x_{2}w+x_{1}\varpi -x_{1}x_{2},$ with the continuous marginal functions $w=1-\bar{w}=F_{{\underline{V}}_{1}}\left(x_{1}\right)\in \left(0,1\right)$ and $\varpi =1-\bar{\varpi }=F_{{\underline{V}}_{1}}\left(x_{2}\right)\in \left(0,1\right)$, where the values $x_{1}$ and $x_{2}$ are in order to guarantee that C(w,ϖ) is of a copula. Then, the associated CDF of the BvPGE-G will be

$$F\left(x_{1},x_{2}\right)=C\left(F_{{\underline{V}}_{1}}\left(x_{1}\right),F_{{\underline{V}}_{1}}\left(x_{2}\right)\right),$$

where $F_{{\underline{V}}_{i}}\left(x_{i}\right)$ is defined above. It is worth mentioning that in [18], the authors emphasize that this copula does not show a closed shape and numerical approaches become necessary.

### 2.3. BvPGE-G Type via FGMCp

Considering the FGMCp (see [10,11,12,13,14,15]), the joint CDF can be written as

$$C_{\Omega }\left(w,\varpi \right)=w\varpi \left(1+\Omega \bar{w}\;\bar{\varpi }\right),$$

where the continuous marginal function is w∈(0,1), ϖ∈(0,1) and Ω∈[−1,1] where $C_{\Omega }\left(w,0\right)=C_{\Omega }\left(0,\varpi \right)=0{|}_{\left(w,\varpi \in \left(0,1\right)\right)},$ which is “grounded minimum condition” and $C_{\Delta }\left(w,1\right)=w$ and $C_{\Delta }\left(1,\varpi \right)=\varpi$, which is “grounded maximum condition”. The grounded minimum/maximum conditions are valid for any copula. Setting $\bar{w}={\bar{w}}_{{\underline{V}}_{1}}{|}_{{\underline{V}}_{1}>0}$ and $\bar{\varpi }={\bar{\varpi }}_{{\underline{V}}_{2}}{|}_{{\underline{V}}_{2}>0}$, then we have

$$F\left(x_{1},x_{2}\right)=C\left(F_{{\underline{V}}_{1}}\left(x_{1}\right),F_{{\underline{V}}_{2}}\left(x_{2}\right)\right)=w\varpi \left(1+\Omega \bar{w}\;\bar{\varpi }\right).$$

The joint PDF can be derived from

$$c_{\Omega }\left(w,\varpi \right)=1+\Omega w^{*}\varpi^{*},\left(w^{*}=1-2w\;and\;\varpi^{*}=1-2\varpi \right)$$

or from

$$f_{\Omega }\left(x_{1},x_{2}\right)=f_{{\underline{V}}_{1}}\left(x_{1}\right)f_{{\underline{V}}_{2}}\left(x_{2}\right)c\left(F_{{\underline{V}}_{1}}\left(x_{1}\right),F_{{\underline{V}}_{2}}\left(x_{2}\right)\right),$$

where the two function $c_{\Omega }\left(w,\varpi \right)$ and $f_{\Omega }\left(x_{1},x_{2}\right)$ are densities corresponding to the joint CDFs $C_{\Omega }\left(w,\varpi \right)$ and $F_{\Omega }\left(x_{1},x_{2}\right)$.

### 2.4. BvPGE-G Type via Modified FGMCp

The modified formula of the modified FGMCp due to [17] can written as

$$C_{\Omega }\left(w,\varpi \right)=w\varpi +\Omega O{\left(w\right)}^{●}\psi {\left(\varpi \right)}^{●},$$

with $O{\left(w\right)}^{●}=w\bar{O\left(w\right)}$ and $\psi {\left(\varpi \right)}^{●}=\varpi \bar{\psi \left(\varpi \right)}$, where O(w)∈(0,1) and ψ(ϖ)∈(0,1) are two continuous functions where O(w=0)=O(w=1)=ψ(ϖ=0)=ψ(ϖ=1)=0. Let

$$\alpha =inf\left\{O{\left(w\right)}^{●}:\partial O{\left(w\right)}^{●},\forall \Delta_{1}\left(w\right)/\partial w\right\}<0,\beta =sup\left\{O{\left(w\right)}^{●}:\partial O{\left(w\right)}^{●},\forall \Delta_{1}\left(w\right)/\partial w\right\}<0,$$

$$\xi =inf\left\{\psi {\left(\varpi \right)}^{●}:\partial \psi {\left(\varpi \right)}^{●},\forall \Delta_{2}\left(\varpi \right)/\partial \varpi \right\}>0,\eta =sup\left\{\psi {\left(\varpi \right)}^{●}:\partial \psi {\left(\varpi \right)}^{●},\forall \Delta_{2}\left(\varpi \right)/\partial \varpi \right\}>0.$$

Then, for 1≤min(βα,ηξ), we have

$$0=\frac{\partial }{\partial w}O{\left(w\right)}^{●}-\frac{w}{\partial w}\partial O\left(w\right)-O\left(w\right),$$

where

$$\Delta_{1}\left(w\right)=\left\{\frac{\partial }{\partial w}O{\left(w\right)}^{●}\;exists\right\},$$

and

$$\Delta_{2}\left(\varpi \right)=\left\{\frac{\partial }{\partial \varpi }\psi {\left(\varpi \right)}^{●}\;exists\right\}.$$

The following four types can be derived and considered:

● ***Type I***

Let ${𝓗}_{1}\left(w\right)\;=\lambda_{1}H_{\theta_{1},\beta_{1},\underline{\xi }}\left(w\right)$ and ${𝓗}_{2}\left(\varpi \right)=\lambda_{2}H_{\theta_{2},\beta_{2},\underline{\xi }}\left(\varpi \right)$. Then, the new bivariate version via modified FGMCp type I can be written as

$$C_{\Omega }\left(w,\varpi \right)=\Omega \left[O{\left(w\right)}^{●}\psi {\left(\varpi \right)}^{●}\;\right]+\left(\begin{array}{c} \left\{{𝓒}_{\lambda_{1}}^{-1}-{𝓒}_{\lambda_{1}}^{-1}exp\left[-{𝓗}_{1}\left(w\right)\right]\right\}\\ \times \left\{{𝓒}_{\lambda_{2}}^{-1}-{𝓒}_{\lambda_{2}}^{-1}exp\left[-{𝓗}_{2}\left(\varpi \right)\right]\right\} \end{array}\right),$$

where

$$O{\left(w\right)}^{●}=w\left\{1-{𝓒}_{\lambda_{1}}^{-1}\left[1-exp\left(-{𝓗}_{1}\left(w\right)\;\right)\right]\right\}{|}_{{\underline{V}}_{1}>0}$$

and

$$\psi {\left(\varpi \right)}^{●}\;=\varpi \left\{1-{𝓒}_{\lambda_{2}}^{-1}\left[1-exp\left(-{𝓗}_{2}\left(\varpi \right)\right)\right]\right\}{|}_{{\underline{V}}_{2}>0}.$$

● ***Type II***

Consider $𝓐\left(w;\Omega_{1}\right)$ and $𝓩\left(\varpi ;\Omega_{2}\right)$ that satisfy the above conditions where $𝓐\left(w;\Omega_{1}\right){|}_{\left(\Omega_{1}>0\right)}=w^{\Omega_{1}}{\left(1-w\right)}^{1-\Omega_{1}}\;\mathrm{and}\;𝓩\left(\varpi ;\Omega_{2}\right){|}_{\left(\Omega_{2}>0\right)}=\varpi^{\Omega_{2}}{\left(1-\varpi \right)}^{1-\Omega_{2}}.$ Then, the corresponding bivariate version (modified FGMCp **Type II**) can be derived from

$$C_{\Omega_{0},\Omega_{1},\Omega_{2}}\left(w,\varpi \right)=w\varpi \left[1+\Omega_{0}𝓐\left(w;\Omega_{1}\right)𝓩\left(\varpi ;\Omega_{2}\right)\right].$$

● ***Type III***

Let $\tilde{𝓐\left(w\right)}=w\left[log\left(1+\bar{w}\right)\right]{|}_{\left(\bar{w}=1-w\right)}\;\mathrm{and}\;\tilde{𝓩\left(\varpi \right)}=\varpi \left[log\left(1+\bar{\varpi }\right)\right]{|}_{\left(\bar{\varpi }=1-\varpi \right)}.$ Then, the associated CDF of the BvPGE-G-FGM (modified FGMCp **Type III**) can be written as

$$C_{\Omega }\left(w,\varpi \right)=w\varpi \left[1+\Omega \tilde{𝓐\left(w\right)}\tilde{𝓩\left(\varpi \right)}\right].$$

● ***Type IV***

Using the quantile concept, the CDF of the BvPGE-G-FGM (modified FGMCp **Type IV**) model can be obtained using

$$C\left(w,\varpi \right)=wF^{-1}\left(w\right)+\varpi F^{-1}\left(\varpi \right)-F^{-1}\left(w\right)F^{-1}\left(\varpi \right)$$

where $F^{-1}\left(w\right)=Q\left(w\right)$ and $F^{-1}\left(\varpi \right)=Q\left(\varpi \right).$

### 2.5. BvPGE-G Type via Ali-Mikhail-Haq Copula

Under the “stronger Lipschitz condition” and following [19], the joint CDF of the Archimedean Ali-Mikhail-Haq copula can written as

$$C_{\Omega }\left(\upsilon ,\nu \right)=\frac{\upsilon \nu }{1-\Omega \bar{\upsilon \nu }}{|}_{\Omega \in \left(-1,1\right)},$$

and the corresponding joint PDF of the Archimedean Ali-Mikhail-Haq copula can be expressed as

$$c_{\Omega }\left(\upsilon ,\nu \right)=\frac{1-\Omega +2\Omega \frac{\upsilon \nu }{1-\Omega \bar{\upsilon \nu }}}{{\left[1-\Omega \bar{\upsilon \nu }\right]}^{2}}{|}_{\Omega \in \left(-1,1\right)},$$

Then, for any $\bar{\upsilon }=1-F_{{\underline{V}}_{1}}\left(x_{1}\right)={|}_{\left[\bar{\upsilon }=\left(1-\upsilon \right)\in \left(0,1\right)\right]}$ and $\bar{\nu }=1-F_{{\underline{V}}_{2}}\left(x_{2}\right){|}_{\left[\bar{\nu }=\left(1-\nu \right)\in \left(0,1\right)\right]},$ we have

$$C_{\Omega }\left(x_{1},x_{2}\right)=\frac{F_{{\underline{V}}_{1}}\left(x_{1}\right)F_{{\underline{V}}_{2}}\left(x_{2}\right)}{1-\Omega \left[1-F_{{\underline{V}}_{1}}\left(x_{1}\right)\right]\left[1-F_{{\underline{V}}_{2}}\left(x_{2}\right)\right]}{|}_{\Omega \in \left(-1,1\right)}$$

$$c_{\Omega }\left(x_{1},x_{2}\right)=\frac{1-\Omega +2\Omega \left\{\frac{F_{{\underline{V}}_{1}}\left(x_{1}\right)F_{{\underline{V}}_{2}}\left(x_{2}\right)}{1-\Omega \left[1-F_{{\underline{V}}_{1}}\left(x_{1}\right)\right]\left[1-F_{{\underline{V}}_{2}}\left(x_{2}\right)\right]}\right\}}{{\left\{1-\Omega \left[1-F_{{\underline{V}}_{1}}\left(x_{1}\right)\right]\left[1-F_{{\underline{V}}_{2}}\left(x_{2}\right)\right]\right\}}^{2}}{|}_{\Omega \in \left(-1,1\right)}$$

## 3. Properties

### 3.1. Expanding the Univariate PDF

In this subsection, we present a useful representation for the new PDF in (4). Based on the new representation, we can easily and directly derive the main statistical properties of the new family due to the exponentiated G (exp-G) family. Using the power series, we expand the quantity $A_{\lambda ,\theta ,\beta }\left(x\right)$. Then, the PDF in (4) can be expressed as

(6) $$f_{\underline{V}}\left(x\right)={𝓒}_{\lambda }^{-1}\beta \theta \overset{+\infty }{\underset{𝓱=0}{\sum }}\frac{{\left(-1\right)}^{𝓱}\lambda^{1+𝓱}g_{\underline{\xi }}\left(x\right)}{𝓱!{\bar{G}}_{\underline{\xi }}{\left(x\right)}^{2}{Ϛ}_{\beta ,\underline{\xi }}\left(x\right)}\underset{B_{\theta \left(𝓱+1\right),\beta }\left(x\right)}{\underbrace{{\left[1-{Ϛ}_{\beta ,\underline{\xi }}\left(x\right)\right]}^{\theta \left(𝓱+1\right)-1}}}$$

Considering the power series

(7) $${\left(1-\frac{z_{1}}{z_{2}}\right)}^{z_{3}+1}=\overset{+\infty }{\underset{𝒾=0}{\sum }}\frac{{\left(-1\right)}^{𝒾}\Gamma \left(z_{3}+2\right)}{𝒾!\Gamma \left(z_{3}-𝒾+2\right)}{\left(\frac{z_{1}}{z_{2}}\right)}^{𝒾},\left|\frac{z_{1}}{z_{2}}\right|\langle 1\;\mathrm{and}\;z_{3}\rangle 0,$$

and applying (7) to the quantity $B_{\theta \left(𝓱+1\right),\beta }\left(x\right)$ in (6), we get

(8) $$f_{\underline{V}}\left(x\right)={𝓒}_{\lambda }^{-1}\beta \theta \frac{g_{\underline{\xi }}\left(x\right)}{{\bar{G}}_{\underline{\xi }}{\left(x\right)}^{2}}\overset{+\infty }{\underset{𝓱,𝒾=0}{\sum }}\lambda^{1+𝓱}\frac{{\left(-1\right)}^{𝓱+𝒾}\Gamma \left(\theta \left(𝓱+1\right)\right)}{𝒾!𝓱!\Gamma \left(\theta \left(𝓱+1\right)-i\right)}\underset{C_{\beta \left(Ϛ+1\right)}\left(x\right)}{\underbrace{exp\left[-\left(𝒾+1\right)\beta \Delta_{\underline{\xi }}\left(x\right)\right]}}$$

Expanding $C_{\beta \left(𝒾+1\right)}\left(x\right)$, we can write

$$C_{\beta \left(𝒾+1\right)}\left(x\right)=\overset{+\infty }{\underset{s=0}{\sum }}{\left(-1\right)}^{s}{\left(𝒾+1\right)}^{s}\frac{G_{\underline{\xi }}{\left(x\right)}^{s}}{\Gamma \left(s+1\right){\bar{G}}_{\underline{\xi }}{\left(x\right)}^{s}}.$$

Inserting the above expression of $C_{\beta \left(𝒾+1\right)}\left(x\right)$ in (8), the PGE-G density reduces to

(9) $$f_{\underline{V}}\left(x\right)=\theta \beta {𝓒}_{\lambda }^{-1}\;\overset{+\infty }{\underset{𝓱,𝒾,\kappa =0}{\sum }}\lambda^{1+𝓱}{\left(-1\right)}^{𝓱+\kappa +𝒾}\frac{\Gamma \left(\theta \left(𝓱+1\right)\right){\left(𝒾+1\right)}^{\kappa }}{𝓱!𝒾!\kappa !\Gamma \left(\theta \left(𝓱+1\right)-𝒾\right)}\frac{g_{\underline{\xi }}\left(x\right)G_{\underline{\xi }}{\left(x\right)}^{\kappa }}{{\bar{G}}_{\underline{\xi }}{\left(x\right)}^{\kappa +2}}.$$

Expanding ${\left[1-G_{\underline{\xi }}\left(x\right)\right]}^{-\kappa -2}$ via generalized binomial expansion, we get

(10) $${\left[1-G_{\underline{\xi }}\left(x\right)\right]}^{-\kappa -2}=\overset{+\infty }{\underset{j=0}{\sum }}\frac{\Gamma \left(1+\kappa^{*}\right)}{j!\Gamma \left(\kappa +2\right)}G_{\underline{\xi }}{\left(x\right)}^{j},\kappa^{*}=\kappa +j+1.$$

Inserting (10) in (9), the PGE-G density can be expressed as

(11) $$f_{\underline{V}}\left(x\right)=\overset{+\infty }{\underset{\kappa ,j=0}{\sum }}\upsilon_{\kappa ,j}g_{\kappa^{*}}\left(x\right),$$

which is an infinite linear combination of exp-G PDFs where $g_{\kappa^{*}}\left(x\right)=dG_{\kappa^{*}}\left(x\right)/dx=\kappa^{*}\pi \left(x\right)G_{\underline{\xi }}{\left(x\right)}^{\kappa +j}$ is the PDF of the exp-G family with power $k^{*}$ and $\upsilon_{\kappa ,j}$ is a constant where

$$\upsilon_{\kappa ,j}=\overset{+\infty }{\underset{𝓱,𝒾=0}{\sum }}\lambda^{1+𝓱}\theta \beta {𝓒}_{\lambda }^{-1}\frac{{\left(-1\right)}^{𝓱+\kappa +𝒾}{\left(𝒾+1\right)}^{\kappa }\Gamma \left(\theta \left(𝓱+1\right)\right)\Gamma \left(1+\kappa^{*}\right)}{𝓱!𝒾!\kappa !j!\kappa^{*}\Gamma \left(\theta \left(𝓱+1\right)-𝒾\right)\Gamma \left(\kappa +2\right)}.$$

Similarly, the CDF of the PGE-G family can also be expressed as

(12) $$F_{\underline{V}}\left(x\right)=\overset{+\infty }{\underset{\kappa ,j=0}{\sum }}\upsilon_{\kappa ,j}\;G_{k^{*}}\left(x\right),$$

where $G_{k^{*}}\left(x\right)$ is the CDF of the exp-G family with power $k^{*}$.

### 3.2. Convex-Concave Analysis

Convex PDFs play a very important role in many areas of mathematics. They are important especially in study of the “optimization problems” where they are distinguished by several convenient properties. In mathematical analysis, a certain PDF defined on a certain n-dimensional interval is called “convex PDF” if the line between any two points on the graph of the PDF lies above the graph between the two points. 

The PDF in (4) and based on any base-line model (see Table 1) is said to be “concave PDF” if for any $X_{1}∼\mathrm{PGE}-G\;\left({\underline{V}}_{1}\right)\;and\;X_{2}∼\mathrm{PGE}-G\;\left({\underline{V}}_{2}\right)$ the PDF satisfies

$$f\left(\Delta x_{1}+\;\bar{\Delta }x_{2}\right)\geq \Delta f_{{\underline{V}}_{1}}\left(x_{1}\right)+\;\bar{\Delta }f_{{\underline{V}}_{2}}\left(x_{2}\right){|}_{0\leq \Delta \leq 1\;\mathrm{and}\;\;\;\bar{\Delta }=1-\Delta }.$$

If the function $f\left(\Delta x_{1}+\bar{\Delta }x_{2}\right)$ is twice differentiable, then if $f^{//}\left(\Delta x_{1}+\;\bar{\Delta }x_{2}\right)<0,\;\forall \;x\in R\;$, $f\left(\Delta x_{1}+\bar{\Delta }x_{2}\right)$ is “strictly convex”. If $f^{//}\left(\Delta x_{1}+\;\bar{\Delta }x_{2}\right)\leq 0,\;\forall \;x\in R$, then $f\left(\Delta x_{1}+\bar{\Delta }x_{2}\right)$ is “convex”. 

The PDF in (4) is said to be “convex PDF” if for any $X_{1}∼\mathrm{PGE}-G\;\left({\underline{V}}_{1}\right)\;and\;X_{2}∼\mathrm{PGE}-G\;\left({\underline{V}}_{1}\right)$ the PDF satisfies

$$f\left(\Delta x_{1}+\;\bar{\Delta }x_{2}\right)\leq \Delta f_{{\underline{V}}_{1}}\left(x_{1}\right)+\;\bar{\Delta }f_{{\underline{V}}_{2}}\left(x_{2}\right){|}_{0\leq \Delta \leq 1\;\mathrm{and}\;\;\;\bar{\Delta }=1-\Delta }.$$

If the function $f\left(\Delta x_{1}+\;\bar{\Delta }x_{2}\right)$ is twice differentiable, then if $f^{//}\left(\Delta x_{1}+\;\bar{\Delta }x_{2}\right)>0,\;\forall \;x\in R\;$, $f\left(\Delta x_{1}+\;\bar{\Delta }x_{2}\right)$ is “strictly convex”. 

If $f^{//}\left(\Delta x_{1}+\;\bar{\Delta }x_{2}\right)\geq 0,\;\forall \;x\in R$, then $f\left(\Delta x_{1}+\;\bar{\Delta }x_{2}\right)$ is “convex”. If $f\left(\Delta x_{1}+\;\bar{\Delta }x_{2}\right)$ is “convex” and c is a constant, then the function $cf\left(\Delta x_{1}+\;\bar{\Delta }x_{2}\right)$ is “convex”. If $f\left(\Delta x_{1}+\;\bar{\Delta }x_{2}\right)$ is “convex PDF”, then $\left[cf\left(\Delta x_{1}+\;\bar{\Delta }x_{2}\right)\right]$ is convex for every c >0. If $f\left(\Delta x_{1}+\;\bar{\Delta }x_{2}\right)$ and $g\left(\Delta x_{1}+\;\bar{\Delta }x_{2}\right)$ are “convex PDF”, then $\left[f\left(\Delta x_{1}+\;\bar{\Delta }x_{2}\right)+g\left(\Delta x_{1}+\;\bar{\Delta }x_{2}\right)\right]$ is also “convex PDF”. If $f\left(\Delta x_{1}+\;\bar{\Delta }x_{2}\right)$ and $g\left(\Delta x_{1}+\;\bar{\Delta }x_{2}\right)$ are “convex PDF”, then $\left[f\left(\Delta x_{1}+\;\bar{\Delta }x_{2}\right).g\left(\Delta x_{1}+\;\bar{\Delta }x_{2}\right)\right]$ is also “convex PDF”. 

If the function $-f\left(\Delta x_{1}+\;\bar{\Delta }x_{2}\right)$ is “convex PDF”, then the function $f\left(\Delta x_{1}+\;\bar{\Delta }x_{2}\right)$ is “convex PDF”. If $f\left(\Delta x_{1}+\;\bar{\Delta }x_{2}\right)$ is “concave PDF”, then $\frac{1}{f\left(\Delta x_{1}+\;\bar{\Delta }x_{2}\right)}$ is “convex PDF” if f(x)>0. If $f\left(\Delta x_{1}+\;\bar{\Delta }x_{2}\right)$ is “concave PDF”, $\frac{1}{f\left(\Delta x_{1}+\;\bar{\Delta }x_{2}\right)}$ is “convex PDF” if f(x)<0. If $f\left(\Delta x_{1}+\;\bar{\Delta }x_{2}\right)$ is “concave PDF”, $f^{-1}\left(\Delta x_{1}+\;\bar{\Delta }x_{2}\right)$ is “convex PDF”.

### 3.3. Moments

Let $Y_{\kappa^{*}}^{}$ be an RV having the exp-G family power with $k^{*}$ and X be an RV having the PGE-G family. Then, the *r*th moment of the RV X is $\mu_{r}^{\prime }=E\left(X^{r}\right)=\overset{n}{\underset{k,j=0}{\sum }}\upsilon_{k,j}E\left(Y_{k^{*}}^{r}\right).$ Analogously, the *n*th moment around the arithmetic mean ($\mu_{1}^{\prime }$) of X is

$$M_{n}=E{\left(X-\mu_{1}^{\prime }\right)}^{n}=\overset{n}{\underset{r=0}{\sum }}\overset{+\infty }{\underset{\kappa ,j=0}{\sum }}\upsilon_{\kappa ,j}\left(\begin{array}{c} n\\ r \end{array}\right){\left(-\mu_{1}^{\prime }\right)}^{n-r}E\left(Y_{\kappa^{*}}^{r}\right).$$

### 3.4. Moment-Generating Function (MGF)

We present two formulas for the obtaining the MGF. Clearly, the first formula can be derived from Equation (11) as

$$M_{X}\left(t\right)=\overset{+\infty }{\underset{\kappa ,j=0}{\sum }}\upsilon_{\kappa ,j}M_{\kappa^{*}}\left(t\right),$$

where $M_{\kappa^{*}}\left(t\right)$ is the MGF of the RV $Y_{\kappa^{*}}$. However, the second formula is based on the concept of the quantile function (QF) as

$$M_{X}\left(t\right)=\overset{+\infty }{\underset{\kappa ,j=0}{\sum }}\upsilon_{\kappa ,j}\tau \left(t,1+\kappa^{*}\right),$$

where the integral

$$\tau \left(t,p\right)=\int_{0}^{1}exp\left[tQ_{G}\left(u\right)\right]u^{p}du$$

can be numerically evaluated using the baseline QF, i.e., $Q_{G}\left(u\right)=G^{-1}\left(u\right)$.

### 3.5. Incomplete Moments (IM)

The *s*th IM, say $\phi_{s,X}\left(t\right)$, of the RV X can be derived from (11) as $\phi_{s,X}\left(t\right)=\overset{n}{\underset{k,j=0}{\sum }}\upsilon_{\kappa ,j}I_{s,\kappa^{*}}^{-\infty ,t}\left(t\right)$ where $I_{s,\kappa^{*}}^{-\infty ,t}\left(t\right)=\int_{-\infty }^{t}x^{s}g_{\kappa^{*}}\left(x\right)dx$. One of the main mathematical end economical applications of the first IM concerns “mean deviations (MD)” and “Bonferroni and Lorenz curves”, which are very useful in economics, insurance, demography, reliability, and medicine. The MD about the $\mu_{1}^{\prime }$ of $E\left(\left|X-\mu_{1}^{\prime }\right|\right)=a_{1}$, and the MD about the median (M) of $E\left(\left|X-M\right|\right)=a_{2}$ of the RV X are given by $a_{1}=2\mu_{1}^{\prime }F\left(\mu_{1}^{\prime }\right)-2\phi_{1,X}\left(\mu_{1}^{\prime }\right)$ and $a_{2}=\mu_{1}^{\prime }-2\phi_{1,X}\left(M\right)$, respectively, where $\mu_{1}^{\prime }=E\left(X\right)\;\mathrm{is}\;\mathrm{the}\;\mathrm{arithmetic}\;\mathrm{mean}\;\mathrm{of}\;\mathrm{the}\;\mathrm{RV}\;X$, M=Q(0.5) is the median of the RV X, and $\phi_{1,X}\left(t\right)$ is the first IM given by $\phi_{s=1,X}\left(t\right)$. Now, we provide two ways to determine $a_{1}$ and $a_{2}$. First, $\phi_{1,X}\left(t\right)=\overset{n}{\underset{\kappa ,j=0}{\sum }}\upsilon_{\kappa ,j}I_{1,\kappa^{*}}\left(t\right)$ where $I_{1,\kappa^{*}}^{-\infty ,t}\left(t\right)$ is the first IM of the exp-G family. Second, $\phi_{1,X}\left(t\right)=\sum_{\kappa ,j=0}^{n}\upsilon_{\kappa ,j}\omega_{\kappa^{*}}\left(t\right)$ where $\omega_{\kappa^{*}}\left(t\right)=\kappa^{*}\int_{0}^{G\left(t\right)}Q_{G}\left(u\right)u^{\kappa^{*}}du$ can be evaluated numerically. 

These results for $\phi_{1,X}\left(t\right)$ can be directly applied for calculating the Bonferroni and Lorenz curves defined, for a certain given probability 𝓟, by $𝓑\left(𝓟\right)=\phi_{1,X}\left(Q\left(𝓟\right)\right)/\left(𝓟\mu_{1}^{\prime }\right)$ and $𝓛\left(𝓟\right)=\phi_{1,X}\left(Q\left(𝓟\right)\right)/\mu_{1}^{\prime }$, respectively. 

### 3.6. Residual Life (RL) and Reversed Residual Life (RRL)

The $q^{th}$ moment of the RL of the RV X can be obtained from $m_{q,X}\left(t\right)=E[\left(X-t{)}^{q}\;\right]{|}_{X>t\;and\;q\in N}$ or from

$$m_{q,X}\left(t\right)=\frac{1}{1-F_{\underline{V}}\left(t\right)}\int_{t}^{\infty }{\left(-t+x\right)}^{q}f_{\underline{V}}\left(x\right)dx,$$

which can also be written as

$$m_{q,X}\left(t\right)=\frac{1}{1-F_{\underline{V}}\left(t\right)}\overset{+\infty }{\underset{\kappa ,j=0}{\sum }}\sum_{h=0}^{q}\upsilon_{\kappa ,j}(\begin{array}{c} q\\ h \end{array}){\left(-t\right)}^{q-h}I_{q,\kappa^{*}}^{-\infty ,t}\left(t\right).$$

For q=1, we obtain the mean of the residual life (MRL) also called the life expectation (LE), which can be drived from $m_{1,X}\left(t\right)=E\left[\left(X-t\right)\right]{|}_{X>t\;and\;q\in N}$ and represents the additional expected life for a certin system or component that is already alive at the age t. On the other hand, the $q^{th}$ moment of the RRL is $M_{q,X}\left(t\right)=E\left[{\left(t-X\right)}^{q}\right]{|}_{X\leq t,\;t>0\;\mathrm{and}\;q\in N}$ or

$$M_{q,X}\left(t\right)=\frac{1}{F_{\underline{V}}\left(t\right)}\int_{0}^{t}{\left(-x+t\right)}^{q}f_{\underline{V}}\left(x\right)dx,$$

which can also be expressed as

$$M_{q,X}\left(t\right)=\frac{1}{F_{\underline{V}}\left(t\right)}\overset{+\infty }{\underset{\kappa ,j=0}{\sum }}\sum_{h=0}^{q}\upsilon_{\kappa ,j}{\left(-1\right)}^{h}\left(\begin{array}{c} q\\ h \end{array}\right)t^{q-h}I_{q,\kappa^{*}}^{-\infty ,t}\left(t\right).$$

For q=1, we obtain the mean waiting time (MWT), which is also called the mean inactivity time (MIT), which can be derived from $M_{1,X}\left(t\right)=E\left[\left(t-X\right)\right]{|}_{X\leq t,\;t>0\;\mathrm{and}\;q=1}$.

### 3.7. Mathematical Results and Numerical Analysis for Two Special Models

We present some mathematical results for two special models chosen from Table 1. All results listed in Table 2 were derived based on the mathematical results previously obtained in Section 1, Section 2, Section 3, Section 4, Section 5 and Section 6. Table 2 (part I) gives mathematical results for the PGEPII model. Table 2 (part II) gives the mathematical results for the PGEE model. The calculations of this Subsection involve several special functions, including the complete beta function

$$B\left(v_{1},v_{2}\right)=\int_{0}^{1}u^{v_{1}-1}{\left(1-u\right)}^{v_{2}-1}du;$$

the incomplete beta function

$$B_{y}\left(v_{1},v_{2}\right)=\int_{0}^{y}u^{v_{1}-1}{\left(1-u\right)}^{v_{2}-1}du,$$

the complete gamma function

$$\begin{array}{cc} \Gamma \left(1+v_{1}\right)= & \int_{0}^{+\infty }t^{v_{1}}exp\left(-t\right)dt=v_{1}!=\\ & \prod_{m=0}^{v_{1}-1}\left(v_{1}-m\right), \end{array}$$

the lower incomplete gamma function

$$\begin{array}{c} \gamma \left(v_{1},v_{2}\right){|}_{\left(v_{1}\neq 0,-1,-2,\ldots \right)}=\int_{0}^{v_{2}}t^{v_{1}-1}exp\left(-t\right)dt\\ =\overset{+\infty }{\underset{v_{3}=0}{\sum }}\frac{{\left(-1\right)}^{v_{3}}}{v_{3}!\left(v_{1}+v_{3}\right)}v_{2}^{v_{1}+v_{3}}, \end{array}$$

and the upper incomplete gamma function

$$\Gamma \left(v_{1},v_{2}\right)=\Gamma \left(v_{1}\right)-\gamma \left(v_{1},v_{2}\right).$$

## 4. Numerical Analysis for Some Measures

Table 3 below gives numerical analysis for the mean (E(X)), variance (V(X)), skewness (S(X)), and kurtosis (K(X)) for PGEPII model based on special case number 7 of Table 1 with a=1. Based on results listed in Table 3, it is noted that E(X) decreases as λ increases, S(X) ∈ (0.647392, ∞) and K(X) ranging from 5.07 to ∞.

## 5. Estimation Method and Assessment

### 5.1. The Maximum Likelihood Estimation (MLE) Method

Let $x_{1},\ldots ,x_{n}$ be an observed random sample (RS) from the PGE-G family with $\underline{V}={\left(\lambda ,\theta ,\beta ,{\underline{\xi }}^{T}\right)}^{T}$. The function of the log-likelihood (${𝓵}_{\underline{V}}=\log\left[\overset{n}{\underset{i=1}{\prod }}f_{\underline{V}}\left(x_{i}\right)\right]$) can be obtained and maximized directly using the R software (the “optim function”) or the program of Ox (sub-routine of MaxBFGS) or MATH-CAD software or by solving the nonlinear equations of the likelihood derived from differentiating ${𝓵}_{\underline{V}}$. The score vector components $U_{\lambda }=\frac{\partial }{\partial \lambda }{𝓵}_{\underline{V}},U_{\theta }=\frac{\partial }{\partial \theta }{𝓵}_{\underline{V}},U_{\beta }=\frac{\partial }{\partial \beta }{𝓵}_{\underline{V}},$ and $U_{{\underline{\xi }}_{k}}=\frac{\partial }{\partial {\underline{\xi }}_{k}}{𝓵}_{\underline{V}}$ can be easily derived from obtaining the nonlinear system $U_{\lambda }=U_{\theta }=U_{\beta }=U_{{\underline{\xi }}_{k}}=0$ and then simultaneously solving them for getting the MLE of $\underline{V}$. This system could be solved numerically for the complicated models using iterative algorithms such as the “Newton–Raphson” algorithms. We can compute the maximum values of the unrestricted and restricted log-likelihoods to obtain likelihood ratio (LR) statistics for testing some sub models. Hypothesis tests of the type $H_{0}:\Omega =\Omega_{0}$ versus $H_{1}:\;\Omega \neq \Omega_{0}$, where Ω is a vector formed with some components of $\underline{V}$ and $\Omega_{0}$ is a specified vector, can be performed using LR statistics. For example, the test of $H_{0}:\lambda =\theta =\beta =1$ versus $H_{1}:\;H_{0}$ is not true and is equivalent to comparing the PGE-G and G distributions, and the LR statistic is given by $W_{LR}=2\left\{{𝓵}_{\underline{V}}\left(\hat{\lambda },\hat{\theta },\hat{\beta },\hat{{\underline{\xi }}^{T}}\right)-𝓵\left(1,1,1,\hat{{\underline{\xi }}^{T}}\right)\right\},$ where $\hat{\lambda },\hat{\theta },\hat{\beta }$ and $\hat{{\underline{\xi }}^{T}}$ are the MLEs under H and $\hat{{\underline{\xi }}^{T}}$ is the estimate under $H_{0}$. 

### 5.2. Graphical Assessment

We present a graphical simulation for assessing the behavior of the finite sample of the MLEs for the PGEPII distribution. We maximized the log-likelihood function using a wide range of starting initial values. The starting initial values were taken in a fine scale. For the PGEPII model, for example, they were taken corresponding to all possible combinations of λ=1,2,…,100,θ=1,2,…,100,β=1,2,…,100,and c=1,2,…,100. The proposed assessment is performed depending on the following assessing algorithm:

Using the QF of the PGEPII distribution, we generate 1000 samples of size n from the PGEPII and PGEE models where

$$Q_{U}={\left(1-\frac{1}{\beta }ln\left\{1-{\left[-\frac{1}{\lambda }\ln\left(1-U{𝓒}_{\lambda }\right)\right]}^{\frac{1}{\theta }}\right\}\right)}^{\frac{1}{c}}-1$$

Computing the standard errors (SEs) of the MLEs for the N = 1000 samples, SEs are obtained via inverting the “observed information matrix”.

Computing the corresponding biases and mean squared errors (MSEs) given for $\underline{V}=\left(\lambda ,\theta ,\beta ,c\right)$, we repeated these steps for n=100, 200,…, 500.

**Figure 3:**
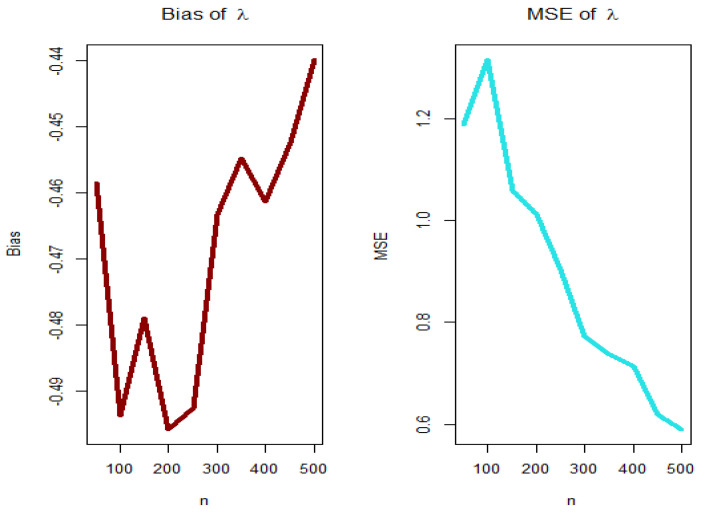
Biases (**left plot**) and mean squared errors (MSEs) (**right plot**) for parameter λ (PGEPII model).

**Figure 4:**
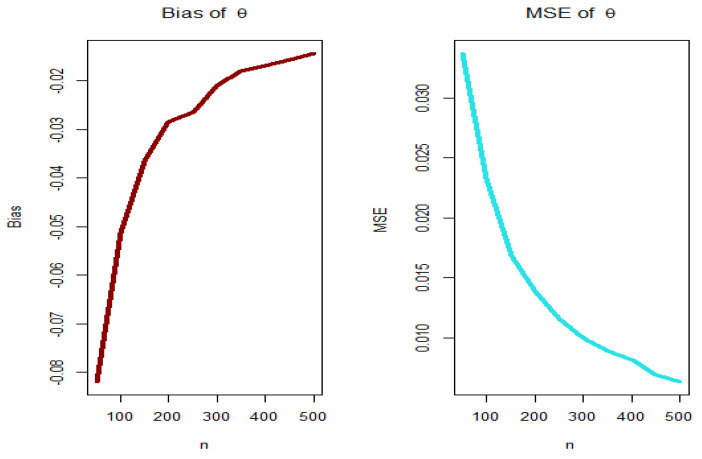
Biases (**left plot**) and MSEs (**right plot**) for parameter θ (PGEPII model).

**Figure 5:**
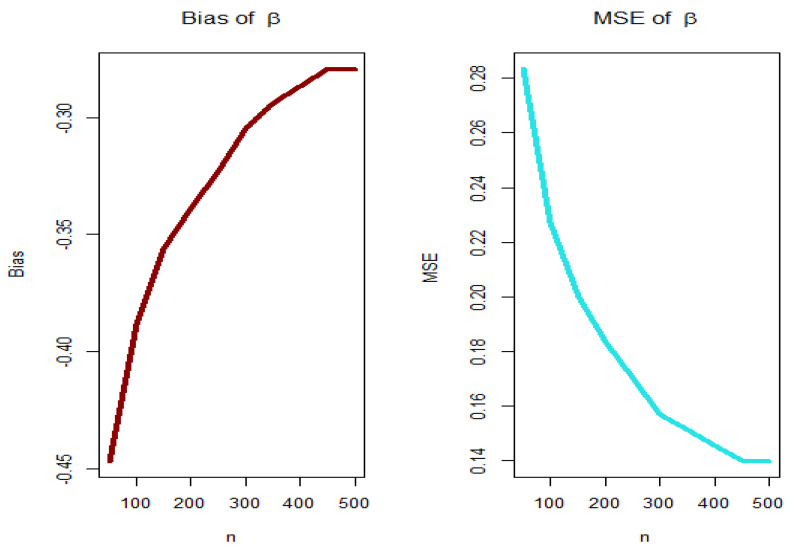
Biases (**left plot**) and MSEs (**right plot**) for parameter β (PGEPII model).

**Figure 6:**
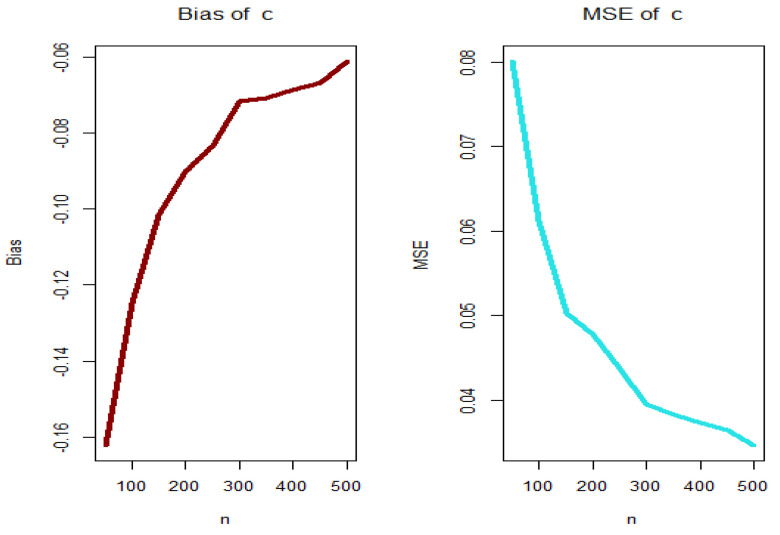
Biases (**left plot**) and MSEs (**right plot**) for parameter c (PGEPII model).

For PGEPII model, Figure 3, Figure 4, Figure 5 and Figure 6 (left panels) show how the four biases vary with respect to n. Figure 3, Figure 4, Figure 5 and Figure 6 (right panels) show how the four MSEs vary with respect to n. From Figure 3, Figure 4, Figure 5 and Figure 6, the biases for each parameter are generally negative and increase to zero as n→∞, and the MSEs for each parameter decrease to zero as n→∞.

## 6. Modeling Failure and Service Times

Two real-life data applications to illustrate the importance and flexibility of the family are presented under the PII case. The fits of the PGEPII are compared with other PII models shown in Table 4. 

**Figure 7:**
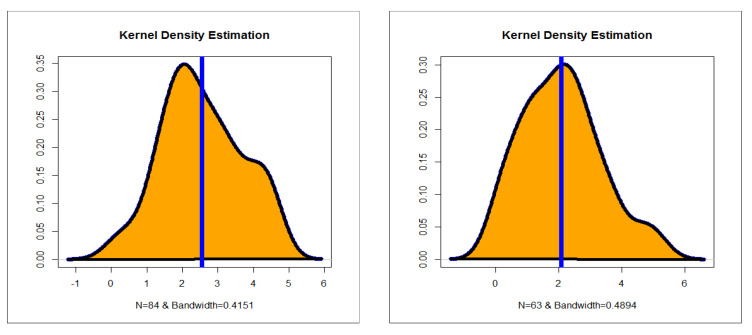
Nonparametric Kernel density estimation (KDE) (1st and 2nd datasets).

**Figure 8:**
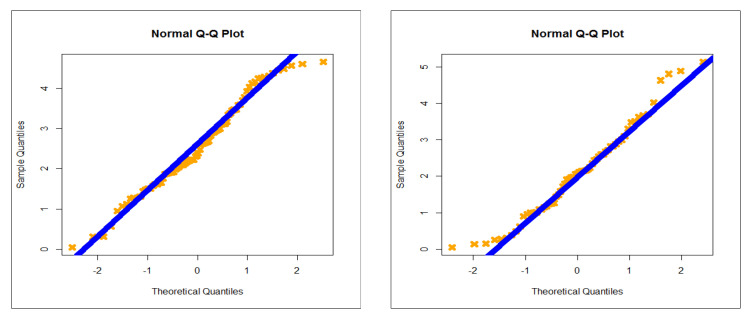
Normal quantile-quantile (Q-Q) plots (1st and 2nd datasets, respectively).

**Figure 9:**
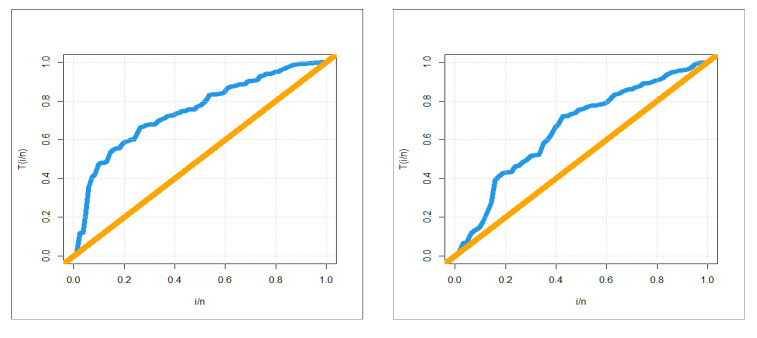
Total time in test (TTT) plots (1st and 2nd datasets, respectively).

**Figure 10:**
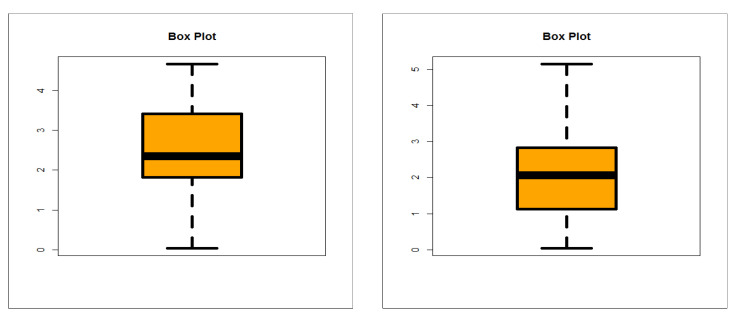
Box plots (1st and 2nd datasets, respectively).

The first dataset (aircraft windshield, n = 84): The first real-life dataset represents the data on failure times of 84 aircraft windshield. The second dataset (aircraft windshield, n = 63): The second real-life dataset represents the data on service times of 63 aircraft windshield. The two real-life datasets were chosen based on matching their characteristics and the plots of the PDF in Figure 1 (the right panel). By examining Figure 1 (the right panel), it is noted that the new PDF can be asymmetric right-skewed function” and “symmetric” with different shapes. On the other hand, by exploring the two real datasets, it is noted that densities are nearly symmetric functions. Additionally, the HRF of the new family includes the asymmetric monotonically increasing shape, and the HRF of the two real datasets are asymmetric monotonically increasing (see Figure 1(left panel)). The two real datasets were reported by [20]. Many other symmetric and asymmetric useful real-life datasets can be found in [21,22,23,24,25,26,27,28]. Initial density shape is explored using the nonparametric “Kernel density estimation (KDE)” approach in Figure 7. The “normality” condition is checked via the “quantile–quantile (Q-Q) plot” in Figure 8. The initial shape of the empirical HRFs is discovered from the “total time in test (TTT)” plot in Figure 9. The extremes are spotted from the “box plot” in Figure 10. Based on Figure 7, it is noted that the densities are nearly symmetric functions. Based on Figure 8, we see that the “normality” nearly exists. Based on Figure 9, it is noted that the HRF is “asymmetric monotonically increasing shaped” for the two datasets. Based on Figure 10, it is showed that no extreme observations were founded. The goodness-of-fit (GOF) statistic called “Akaike information” (AICr), consistent-AIC (CAICr), Bayesian-IC (BICr), and Hannan–Quinn-IC (HQICr) were analyzed by comparing the competitive PII models. 

**Table 4:** The competitive models.

N.	Model	Abbreviation	Author
1	Special generalized mixture-PII	SGMPII	[29]
2	Odd log-logistic-PII	OLLPII	[30]
3	Reduced OLL-PII	ROLLPII	[30]
4	Reduced Burr–Hatke-PII	RBHPII	[31]
5	Transmuted Topp–Leone-PII	TTLPII	[32]
6	Reduced TTL-PII	RTTLPII	[32]
7	Gamma-PII	GamPII	[33]
8	Kumaraswamy-PII	KumPII	[34]
9	McDonald-PII	McPII	[34]
10	Beta-PII	BPII	[34]
11	Exponentiated-PII	EPII	[35]
12	PII	PII	[36]
13	Proportional reversed hazard rate PII	PRHRPII	New

**Figure 11:**
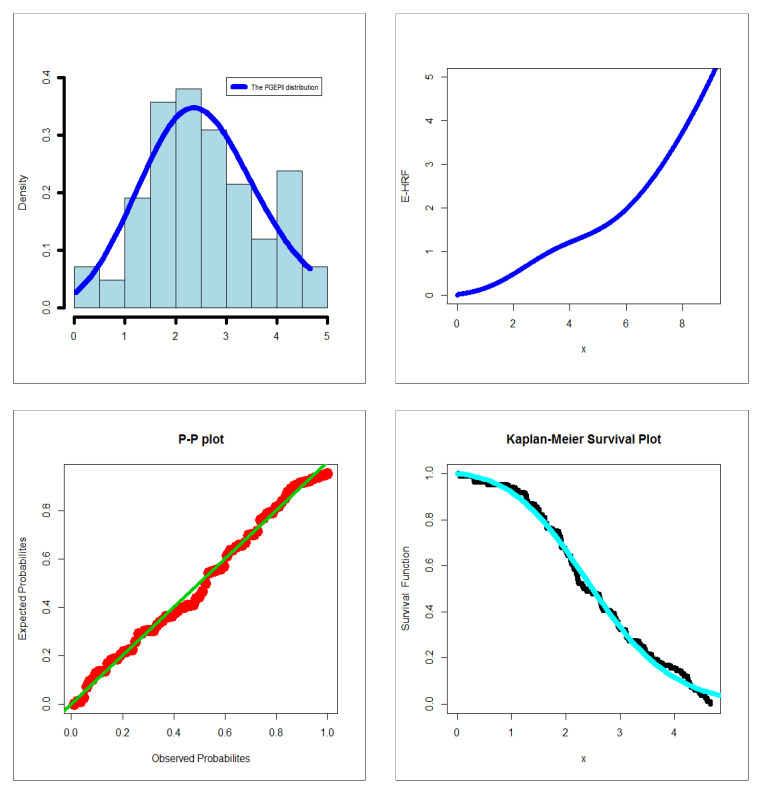
Estimated PDF (EPDF), estimated HRF (EHRF), probability–probability (P-P), and Kaplan-Meier survival (KMS) plots for the 1st dataset.

**Figure 12:**
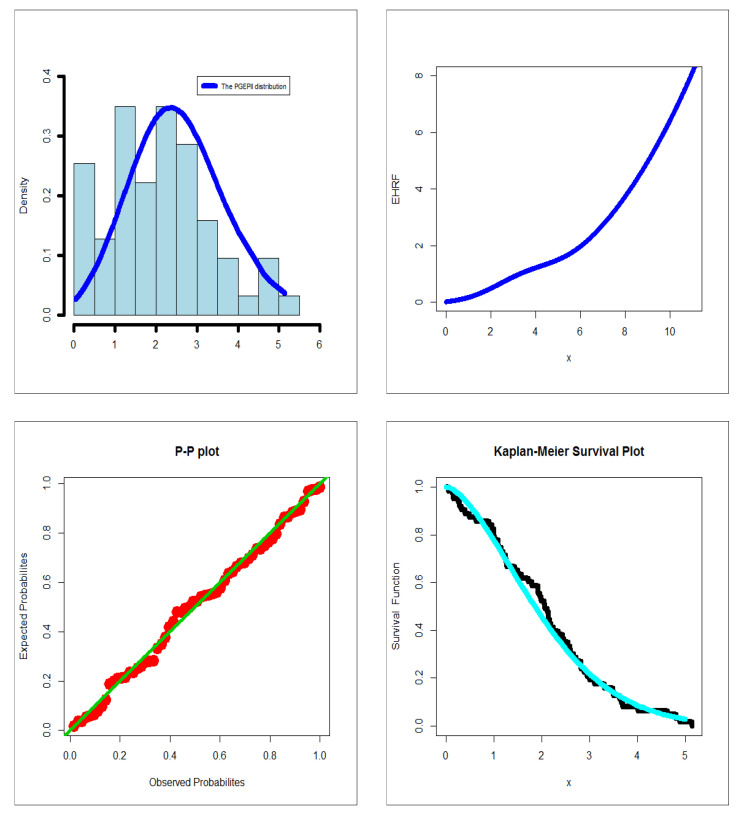
EPDF, EHRF, P-P, and KMS plots for the 2nd dataset.

However, many other PII extensions could be considered in comparisons [37,38,39,40,41,42,43,44,45]. For failure times real-life data, relevant numerical results are shown in Table 5 and Table 6. Precisely, Table 5 gives the MLEs and SEs. Table 6 gives the four GOF test statistics. For service times real-life data, the results are presented in Table 7 and Table 8. Precisely, Table 5 gives the MLEs and SEs, whereas Table 8 gives the four GOFs test statistics. Figure 11 and Figure 12 give the probability-probability (P-P) plot, estimated PDF (EPDF), Kaplan-Meier survival (KMS) plot and estimated HRF (EHRF) plot for the two datasets, respectively. Based on Table 6 and Table 8, it is noted that the PGEPII model gives the lowest values for all test statistics, where AICr = 264.231, CAICr = 264.737, BICr = 273.954, and HQICr = 268.139 for the first dataset, and AICr = 205.252, CAICr = 205.941, BICr = 213.824, and HQICr = 208.623 for the second dataset among all fitted models. Hence, it could be chosen as the best model under these criteria.

Further, the results of the LR statistics of the PGEPII model against the quasi-Poisson generalized exponential Pareto type II (QPGEPII), Poisson exponential Pareto type II (PEPII), and quasi-Poisson Pareto type II (QPPII) models under the first dataset are in Table 9. Based on the results of this table,

**I**-We reject the null hypotheses of the LR tests in favor of the PGEPII model. 

**II**-We can confirm the significance of the parameters λ and θ with $W_{LR}=17.09761$**,** $W_{LR}=14.27654,$ and $W_{LR}=9.00651,$ respectively.

The results of the LR statistics of the PGEPII model against the QPGEPII, PEPII, and QPPII models under the second dataset are in Table 10. Based on the results of this table,

**I**-We reject the null hypotheses of the LR tests in favor of the PGEPII model. 

**II**-We can confirm the significance of the parameters λ and θ with $W_{LR}=33.01982$**,** $W_{LR}=4.710811$, and $W_{LR}=3.476109$, respectively.

## 7. Conclusions

In this article, a new parametric lifetime compound G family of continuous probability distributions called the Poisson generalized exponential G (PGEG) family is derived and studied. The PGEG family is defined based on the Poisson and the generalized exponential G families’ concept of compounding. The new density can be “asymmetric right-skewed function”, “asymmetric left-skewed”, “bimodal”, and “symmetric” with different shapes. The new HRF can be “upside down bathtub”, “bathtub”, “decreasing-constant”, “increasing-constant”, “increasing”, “constant“, and “increasing”. Relevant mathematical properties including moments, incomplete moments, and mean deviation are derived. Some new bivariate-type PGEG families using the “copula of Farlie-Gumbel-Morgenstern”, “copula of the modified Farlie-Gumbel-Morgenstern”, “the Clayton copula”, and “copula Renyi’s entropy” are presented. Many special members are derived, and special attention is devoted to the exponential (E) and the one parameter Pareto type II (PII) model. A simulation study is presented to assess the finite sample behavior of the estimators. The simulations are based on a certain given algorithm under the baseline PII model. Finally, two different real-life applications are proposed to illustrate the importance of the PGEG family. For all real data, for exploring the “initial shape”, the nonparametric Kernel density estimation is presented. For checking the “normality” condition, the “Quantile–Quantile plot” is presented. For discovering the shape of the HRFs, the “total time in test” plot is provided. To explore the extremes, the “box plot” is sketched. Based on PII base-line model, the PEWPII model gives the lowest values for all test statistics, where AICr = 264.231, CAICr = 264.737, BICr = 273.954, and HQICr = 268.139 for the failure times data; AICr = 205.252, CAICr = 205.941, BICr = 213.824, and HQICr = 208.623 for the service times data.

## Figures and Tables

**Table 1:** Some new members derived based on the Poisson generalized exponential G (PGEG) family.

No.	Baseline Model	**$\Delta_{\underline{\xi }}\left(x\right)$**	V = $\left(\lambda ,\theta ,\beta ,\underline{\xi }\right)$	New Model
1	Exponential (E)	exp(cx)−1	$\lambda \in R-\left\{0\right\},\theta >0,\beta >0,\underline{\xi }=(c>0)$	PGEE
2	Log-logistic (LL)	${\left(\frac{x}{a}\right)}^{c}$	$\lambda \in R-\left\{0\right\},\theta >0,\beta >0,\underline{\xi }=(a,c>0)$	PGELL
3	Weibull (W)	$exp{\left(ax\right)}^{c}-1$	$\lambda \in R-\left\{0\right\},\theta >0,\beta >0,\underline{\xi }=(a,c>0)$	PGEW
4	Fréchet (F)	${\left[exp\left(x^{-c}\right)-1\right]}^{-1}$	$\lambda \in R-\left\{0\right\},\theta >0,\beta >0,\underline{\xi }=(c>0)$	PGEF
5	Rayleigh (R)	$exp{\left(cx\right)}^{2}-1$	$\lambda \in R-\left\{0\right\},\theta >0,\beta >0,\underline{\xi }=(c>0)$	PGER
6	Dagum (D)	${\left[{\left(1+{\left(\frac{x}{b}\right)}^{-a}\right)}^{c}-1\right]}^{-1}$	$\lambda \in R-\left\{0\right\},\theta >0,\beta >0,\underline{\xi }=(a,b,c>0)$	PGED
7	Pareto type II (PII)	${\left(1+x/a\right)}^{c}-1$	$\lambda \in R-\left\{0\right\},\theta >0,\beta >0,\underline{\xi }=(a,c>0)$	PGEPII
8	Burr type XII (BXII)	${\left(1+x^{a}\right)}^{c}-1$	$\lambda \in R-\left\{0\right\},\theta >0,\beta >0,\underline{\xi }=(a,c>0)$	PGEBXII
9	Lindley (Li)	$\frac{exp\left(cx\right)}{\left[\frac{1+c+cx}{1+c}\right]}-1$	$\lambda \in R-\left\{0\right\},\theta >0,\beta >0,\underline{\xi }=(c>0)$	PGELi
10	Inverse Rayleigh (IR)	${\left[exp\left(ax^{-2}\right)-1\right]}^{-1}$	$\lambda \in R-\left\{0\right\},\theta >0,\beta >0,\underline{\xi }=(c>0)$	PGEIR
11	Half-logistic (HL)	${\left\{{\left[\frac{1-exp\left(-cx\right)}{1+exp\left(-cx\right)}\right]}^{-1}-1\right\}}^{-1}$	$\lambda \in R-\left\{0\right\},\theta >0,\beta >0,\underline{\xi }=(c>0)$	PGEHL
12	Inverse Exponential (IE)	${\left[exp\left(ax^{-1}\right)-1\right]}^{-1}$	$\lambda \in R-\left\{0\right\},\theta >0,\beta >0,\underline{\xi }=(c>0)$	PGEIE
13	Inverse PII	${\left[{\left(1+ax^{-1}\right)}^{c}-1\right]}^{-1}$	$\lambda \in R-\left\{0\right\},\theta >0,\beta >0,\underline{\xi }=(a,c>0)$	PGEIPII
14	Gumbel (Gu)	${\left(exp\left\{exp\left[-\frac{x-c}{a}\right]\right\}-1\right)}^{-1}$	$\lambda \in R-\left\{0\right\},\theta >0,\beta >0,\underline{\xi }=(a>0,c\in R)$	PGEGu
15	Burr type XII (BXII)	${\left[{\left(1+{\left(x/\lambda \right)}^{a}\right)}^{c}-1\right]}^{-1}$	$\lambda \in R-\left\{0\right\},\theta >0,\beta >0,\underline{\xi }=(a,c>0)$	PGEBXII
16	Fréchet (F)	${\left[exp\left(a^{c}x^{-c}\right)-1\right]}^{-1}$	$\lambda \in R-\left\{0\right\},\theta >0,\beta >0,\underline{\xi }=(a,c>0)$	PGEF
17	Burr type X (BX)	${\left({\left\{1-exp\left[-{\left(ax\right)}^{2}\right]\right\}}^{-c}-1\right)}^{-1}$	$\lambda \in R-\left\{0\right\},\theta >0,\beta >0,\underline{\xi }=(a,c>0)$	PGEBX
18	Standard Gumbel (Gu)	${\left(exp\left\{exp\left[-\left(ax\right)\right]\right\}-1\right)}^{-1}$	$\lambda \in R-\left\{0\right\},\theta >0,\beta >0,\underline{\xi }=(a>0)$	PGESGu
19	Nadarajah-Haghighi (NH)	$exp\left[{\left(1+ax\right)}^{c}-1\right]-1$	$\lambda \in R-\left\{0\right\},\theta >0,\beta >0,\underline{\xi }=(a,c>0)$	PGENH
20	Gompertz	exp{a[exp(cx)−1]}−1	$\lambda \in R-\left\{0\right\},\theta >0,\beta >0,\underline{\xi }=(a,c>0)$	PGEGz
21	Inverse Flexible Weibull (IFW)	${\left(exp\left\{exp\left[a/y-cx\right]\right\}-1\right)}^{-1}$	$\lambda \in R-\left\{0\right\},\theta >0,\beta >0,\underline{\xi }=(a,c>0)$	PGEIFW
22	Inverse Gompertz (IGz)	${\left\{exp\left[-\frac{exp\left(cx\right)-1}{c}\right]-1\right\}}^{-1}$	$\lambda \in R-\left\{0\right\},\theta >0,\beta >0,\underline{\xi }=(c>0)$	PGEIGz
23	Normal (N)	$\frac{\varphi \left(\frac{x-c}{a}\right)}{1-\varphi \left(\frac{x-c}{a}\right)}$	$\lambda \in R-\left\{0\right\},\theta >0,\beta >0,\underline{\xi }=(a>0,c\in R)$	PGEN
24	Gamma (Ga)	$\frac{1}{\Gamma^{-1}\left(a\right)}\gamma^{-1}\left(a,\frac{x}{c}\right)-1$	$\lambda \in R-\left\{0\right\},\theta >0,\beta >0,\underline{\xi }=(a,c>0)$	PGEGa

**Table 2:** Mathematical results for the PGEPII model.

	Part I	
Property	Result	Support
$E\left(X^{r}\right)$	$\overset{+\infty }{\underset{k,j=0}{\sum }}\overset{r}{\underset{\nu =0}{\sum }}\upsilon_{k,j}k^{*}a^{r}{\left(-1\right)}^{\nu }\left(\begin{array}{c} r\\ \nu \end{array}\right)B\left(k^{*},\frac{\nu -r}{c}+1\right)$	c>r
$M_{X}\left(t\right)$	$\overset{+\infty }{\underset{k,j,r=0}{\sum }}\overset{r}{\underset{\nu =0}{\sum }}\frac{t^{r}}{r!}\upsilon_{k,j}k^{*}a^{r}{\left(-1\right)}^{\nu }\left(\begin{array}{c} r\\ \nu \end{array}\right)B\left(k^{*},\frac{\nu -r}{c}+1\right)$	c>r
$\phi_{s,X}\left(t\right)$	$\overset{+\infty }{\underset{k,j=0}{\sum }}\overset{s}{\underset{\nu =0}{\sum }}\upsilon_{k,j}k^{*}a^{s}{\left(-1\right)}^{\nu }\left(\begin{array}{c} s\\ \nu \end{array}\right)B_{t}\left(k^{*},\frac{\nu -s}{c}+1\right)$	c>s
$\phi_{1,X}\left(t\right)$	$\overset{+\infty }{\underset{k,j=0}{\sum }}\overset{1}{\underset{\nu =0}{\sum }}\upsilon_{k,j}k^{*}a{\left(-1\right)}^{\nu }\left(\begin{array}{c} 1\\ \nu \end{array}\right)B_{t}\left(k^{*},\frac{\nu -1}{c}+1\right)$	c>1
$m_{q,X}\left(t\right)$	$\frac{1}{1-F_{\underline{V}}\left(t\right)}\overset{+\infty }{\underset{k,j=0}{\sum }}\overset{q}{\underset{\nu =0}{\sum }}\upsilon_{k,j,v}\left(m,q\right)\;k^{*}a^{q}{\left(-1\right)}^{\nu }\left(\begin{array}{c} q\\ \nu \end{array}\right)B_{t}\left(k^{*},\frac{\nu -q}{c}+1\right),$ where $\upsilon_{k,j,v}\left(m,q\right)=\upsilon_{k,j}\sum_{h=0}^{q}\left(\begin{array}{c} q\\ h \end{array}\right){\left(-t\right)}^{q-h}$	t>0, q∈N, c>q
$m_{1,X}\left(t\right)$	$\frac{1}{1-F_{\underline{V}}\left(t\right)}\overset{+\infty }{\underset{k,j=0}{\sum }}\overset{1}{\underset{\nu =0}{\sum }}\upsilon_{k,j,v}\left(m,1\right)\;k^{*}a{\left(-1\right)}^{\nu }\left(\begin{array}{c} 1\\ \nu \end{array}\right)B_{t}\left(k^{*},\frac{\nu -1}{c}+1\right)$ where $\upsilon_{k,j,v}\left(m,1\right)=\upsilon_{k,j}\sum_{h=0}^{1}\left(\begin{array}{c} 1\\ h \end{array}\right){\left(-t\right)}^{1-h}$	t>0, q=1 c>1
$M_{q,X}\left(t\right)$	$\frac{1}{F_{\underline{V}}\left(t\right)}\overset{+\infty }{\underset{k,j=0}{\sum }}\overset{q}{\underset{\nu =0}{\sum }}\upsilon_{k,j,v}\left(M,q\right)\;k^{*}a^{q}{\left(-1\right)}^{\nu }\left(\begin{array}{c} q\\ \nu \end{array}\right)B_{t}\left(k^{*},\frac{\nu -q}{c}+1\right),$ where $\upsilon_{k,j,v}\left(M,q\right)=\upsilon_{k,j}\sum_{h=0}^{q}{\left(-1\right)}^{h}\left(\begin{array}{c} q\\ r \end{array}\right)t^{q-h}$	t>0, q∈N, c>q
$M_{1,X}\left(t\right)$	$\frac{1}{F_{\underline{V}}\left(t\right)}\overset{+\infty }{\underset{k,j=0}{\sum }}\overset{1}{\underset{\nu =0}{\sum }}\upsilon_{k,j,v}\left(M,1\right)\;k^{*}a{\left(-1\right)}^{\nu }\left(\begin{array}{c} 1\\ \nu \end{array}\right)B_{t}\left(k^{*},\frac{\nu -1}{c}+1\right)$ where $\upsilon_{k,j,v}\left(M,1\right)=\upsilon_{k,j}\sum_{h=0}^{1}{\left(-1\right)}^{h}\left(\begin{array}{c} 1\\ r \end{array}\right)t^{1-h}$	t>0, q=1 c>1
	**Part II**	
**Property**	**Result**	**Support**
$E\left(X^{r}\right)$	$\frac{1}{c^{r}}\Gamma \left(r+1\right)\overset{+\infty }{\underset{k,j,h=0}{\sum }}\upsilon_{k,j}\frac{k^{*}{\left(-1\right)}^{h}}{{\left(h+1\right)}^{-\left(r+1\right)}}\left(\begin{array}{c} k^{*}-1\\ h \end{array}\right)$	r>−1
$M_{X}\left(t\right)$	$\frac{1}{c^{r}}\overset{+\infty }{\underset{k,j,r,h=0}{\sum }}\upsilon_{k,j}\frac{t^{r}k^{*}{\left(-1\right)}^{h}}{{\left(h+1\right)}^{-\left(r+1\right)}}\left(\begin{array}{c} k^{*}-1\\ h \end{array}\right)$	r>−1
$\phi_{s,X}\left(t\right)$	$\frac{1}{c^{s}}\gamma \left(r+1,ct\right)\overset{+\infty }{\underset{k,j,h=0}{\sum }}\upsilon_{k,j}\frac{k^{*}{\left(-1\right)}^{h}}{{\left(h+1\right)}^{-\left(r+1\right)}}\left(\begin{array}{c} k^{*}-1\\ h \end{array}\right)$	s>−1
$\phi_{1,X}\left(t\right)$	$\frac{1}{c}\gamma \left(2,ct\right)\overset{+\infty }{\underset{k,j,h=0}{\sum }}\upsilon_{k,j}\frac{k^{*}{\left(-1\right)}^{h}}{{\left(h+1\right)}^{-2}}\left(\begin{array}{c} k^{*}-1\\ h \end{array}\right)$	s=1
$m_{q,X}\left(t\right)$	$\frac{1}{c^{q}\left[1-F_{\underline{V}}\left(t\right)\right]}\Gamma \left(q+1,ct\right)\overset{+\infty }{\underset{k,j,h=0}{\sum }}\upsilon_{k,j,h}\left(m,q\right)\frac{k^{*}{\left(-1\right)}^{h}}{{\left(h+1\right)}^{-\left(q+1\right)}}\left(\begin{array}{c} k^{*}-1\\ h \end{array}\right)$	t>0, q∈N.
$m_{1,X}\left(t\right)$	$\frac{1}{c\left[1-F_{\underline{V}}\left(t\right)\right]}\Gamma \left(2,ct\right)\overset{+\infty }{\underset{k,j,h=0}{\sum }}\upsilon_{k,j,h}\left(m,1\right)\frac{k^{*}{\left(-1\right)}^{h}}{{\left(h+1\right)}^{-2}}\left(\begin{array}{c} k^{*}-1\\ h \end{array}\right)$	t>0, q=1.
$M_{q,X}\left(t\right)$	$\frac{1}{c^{q}F_{\underline{V}}\left(t\right)}\gamma \left(q+1,ct\right)\overset{+\infty }{\underset{k,j,h=0}{\sum }}\upsilon_{k,j,h}\left(M,q\right)\frac{k^{*}{\left(-1\right)}^{h}}{{\left(h+1\right)}^{-\left(q+1\right)}}\left(\begin{array}{c} k^{*}-1\\ h \end{array}\right)$	t>0, q∈N.
$M_{1,X}\left(t\right)$	$\frac{1}{cF_{\underline{V}}\left(t\right)}\gamma \left(2,ct\right)\overset{+\infty }{\underset{k,j,h=0}{\sum }}\upsilon_{k,j,h}\left(M,1\right)\frac{k^{*}{\left(-1\right)}^{h}}{{\left(h+1\right)}^{-2}}\left(\begin{array}{c} k^{*}-1\\ h \end{array}\right)$	t>0, q=1

**Table 3:** E(X), V(X), S(X), and kurtosis K(X) for PGEPII model.

**λ**	**θ**	**β**	**c**	**E(X)**	**V(X)**	**S(X)**	**K(X)**
−100	10	10	0.5	2.072196	0.2201758	1.479884	7.298747
−50				1.833215	0.2047501	1.485328	7.352612
1				0.602749	0.0926237	1.947101	10.23900
10				0.3201456	0.0086203	0.922245	6.964258
20				4.5 × 10^−7^	4.9 × 10^−7^	1557.789	2427588
50				3 × 10^−18^	3.2 × 10^−18^	∞	∞
1	0.00001	1.5	1.5	3.8 × 10^−6^	1.9 × 10^−6^	617.3573	518800.1
	0.001			0.000382	0.00019439	62.16521	5164.672
	0.1			0.037952	0.01799428	6.116264	52.94105
	1			0.300097	0.09320253	1.923912	8.063683
	10			0.943049	0.11873920	1.095806	5.033141
	200			1.796896	0.09144218	1.094972	5.171026
	500			2.035741	0.08487209	1.113656	5.249637
	1000			2.210426	0.08057697	1.126665	5.304018
	5000			2.598923	0.07236505	1.152185	5.412047
	10,000			2.759814	0.06942454	1.161333	5.451521
	50,000			3.120738	0.06361832	1.179193	5.530603
	10^5^			3.271321	0.06147196	1.185689	5.559284
	10^6^			3.753629	0.05547417	1.203521	5.640401
	10^9^			5.074701	0.04376374	1.236481	5.797372
0.5	10	0.1	0.5	0.556669	45.25801	12.39501	158.3764
		0.5		35.16515	534.9123	**0.647392**	2.897928
		1		14.48305	114.1355	2.361592	11.45837
		10		0.6436296	0.105070	1.824918	9.34089
		50		0.1142242	0.002606	1.477433	6.578002
1.5	1.5	1.5	0.0001	0.0009722	0.052934	296.8286	97854.25
			0.01	0.9289666	49.47247	9.459858	101.0864
			0.5	1.9094220	7.498718	4.979968	50.15636
			1	0.6041312	0.336279	2.300106	11.34566
			2	0.250036	0.041541	1.588718	6.432767
			3	0.1572757	0.014881	1.401211	5.473245
			4	0.1146732	0.007539	1.314559	**5.074107**
			5	0.09022103	0.004537	1.264612	49.73842

**Table 5:** Maximum Likelihood Estimation (MLEs) and standard errors (SEs) for **1st** dataset.

Model	Estimates			
PGEPII (λ,θ,β,c)	**2.82464**	**1.03661**	**0.002702**	**3.69627**
	****(7.4304)****	****(0.07303)****	****(0.00046)****	****(0.0004)****
KPII (θ,β, c,α)	2.61502	100.276	5.27710	78.6774
	(0.3822)	(120.49)	(9.8116)	(186.01)
TTLPII (θ,β, c,α)	−0.80751	2.47663	(15,608)	(38,628)
	(0.1396)	(0.5418)	(1602.4)	(123.94)
BPII (θ,β, c,α)	3.60360	33.6387	4.83070	118.837
	(0.6187)	(63.715)	(9.2382)	(428.93)
PRHRPII (β, c,α)	3.73 × 10^6^	4.17 × 10^−1^	4.51 × 10^6^	
	1.01 × 10^6^	(0.00001)	37.1468	
SGMPII (θ, c,α)	−1.04 × 10^−1^	9.83 × 10^6^	1.18 × 10^7^	
	(0.1223)	(4843.3)	(501.04)	
RTTLPII (θ,β, c)	−0.84732	5.52057	1.15678	
	(0.10011)	(1.1848)	(0.0959)	
OLLPII (θ, c,α)	2.32636	7.17 × 10^5^	2.3 × 10^6^	
	(2.14 × 10^−1^)	(1.19 × 10^4^)	(2.6 × 10^1^)	
EPII (θ, c,α)	3.62610	20,074.5	26,257.7	
	(0.6236)	(2041.8)	(99.744)	
GamPII (θ, c,α)	3.58760	52,001.4	37,029.7	
	(0.5133)	(7955.0)	(81.163)	
ROLLPII (θ, c)	3.89056	0.57316		
	(0.3652)	(0.0195)		
RBHPII (c,α)	1,080,175	513,672		
	(983,309)	(23,231)		
PII (c,α)	51,425.4	131,790		
	(5933.5)	(296.12)		

**Table 6:** Goodness-of-fit (GOF) statistics for **1st** dataset.

Model	AICr	BICr	CAICr	HQICr
**PGEPII**	**264.231**	**273.954**	**264.737**	**268.139**
OLLPII	274.847	282.139	275.147	277.779
TTLPII	279.140	288.863	279.646	283.049
GamPII	282.808	290.136	283.105	285.756
BPII	285.435	295.206	285.935	289.365
EPII	288.799	296.127	289.096	291.747
ROLLPII	289.690	294.552	289.839	291.645
SGMPII	292.175	299.467	292.475	295.106
RTTLPII	313.962	321.254	314.262	316.893
PRHRPII	331.754	339.046	332.054	334.686
PII	333.977	338.862	334.123	335.942
RBHPII	341.208	346.070	341.356	343.162

**Table 7:** MLEs and SEs for **2nd** dataset.

Model	Estimates			
PGEPII (λ,θ,β,c)	**−4.38494**	**0.34355**	**0.10422**	**2.11596**
	**(10.4313)**	**(0.0009)**	**(0.1068)**	**(0.6017)**
BPII (θ,β, c,α)	1.921842	31.2594	4.9684	169.572
	(0.3184)	(316.84)	(50.528)	(339.21)
KPII (θ,β, c,α)	1.66912	60.5673	2.56490	65.0640
	(0.2571)	(86.013)	(4.7589)	(177.59)
TTLPII (θ,β, c,α)	(−0.607)	1.78578	2123.39	4822.79
	(0.2137)	(0.4152)	(163.92)	(200.01)
RTTLPII (θ,β, c)	−0.67151	2.74496	1.01238	
	(0.18746)	(0.6696)	(0.1141)	
PRHRPII (β, c,α)	1.59 × 10^6^	3.93 × 10^−1^	1.30 × 10^6^	
	2.01 × 10^3^	0.0004 × 10^−1^	0.95 × 10^6^	
SGMPII (θ, c,α)	−1.04 × 10^−1^	6.45 × 10^6^	6.33 × 10^6^	
	(4.1 × 10^−10^)	(3.21 × 10^6^)	(3.8573)	
GamPII (θ, c,α)	1.9073232	35,842.433	39,197.57	
	(0.32132)	(6945.074)	(151.653)	
OLLPII (θ, c,α)	1.66419	6.340 × 10^5^	2.01 × 10^6^	
	(1.8 × 10^−1^)	(1.68 × 10^4^)	7.22 × 10^6^	
EPII (θ, c,α)	1.914532	22,971.15	32,882.0	
	(0.34801)	(3209.53)	(162.22)	
RBHPII (c,α)	14,055,522	53,203,423		
	(422.01)	(28.5232)		
ROLLPII (θ, c)	2.372331	0.69109		
	(0.26834)	(0.0449)		
PII (c,α)	99,269.83	207,019.4		
	(11864.3)	(301.237)		

**Table 8:** GOF statistics for **2nd** dataset.

Model	AICr	BICr	CAICr	HQICr
**PGEPII**	**205.252**	**213.824**	**205.941**	**208.623**
KPII	209.735	218.308	210.425	213.107
TTLPII	212.900	221.472	213.589	216.271
GamPII	211.666	218.096	212.073	214.195
SGMPII	211.788	218.218	212.195	214.317
BPII	213.922	222.495	214.612	217.294
EPII	213.099	219.529	213.506	215.628
OLLPII	215.808	222.238	216.215	218.337
PRHRPII	224.597	231.027	225.004	227.126
PII	222.598	226.884	222.798	224.283
ROLLPII	225.457	229.744	225.657	227.143
RTTLPII	230.371	236.800	230.778	232.900
RBHPII	229.201	233.487	229.401	230.887

**Table 9:** The likelihood ratio (LR) statistics for the **1st** dataset.

Model	Hypothesis	$W_{LR}$	*p*-Value
PGEPII vs. QPGEPII	$H_{0}$: λ=1$,\;H_{1}$$:\;H_{0}$ false	17.09761	0.0015
PGEPII vs. PEPII	$H_{0}$: θ=1$,\;H_{1}$$:\;H_{0}$ false	14.27654	0.0122
PGEPII vs. QPPII	$H_{0}$: λ=θ=1$,\;H_{1}$$:\;H_{0}$ false	9.00651	0.0953

**Table 10:** The LR statistics for the **2nd** data.

Model	Hypothesis	$W_{LR}$	*p*-Value
PGEPII vs. QPGEPII	$H_{0}$: λ=1$,\;H_{1}$$:\;H_{0}$ false	33.01982	0.0011
PGEPII vs. PEPII	$H_{0}$: θ=1$,\;H_{1}$$:\;H_{0}$ false	4.710811	0.0033
PGEPII vs. QPPII	$H_{0}$: λ=θ=1$,\;H_{1}$$:\;H_{0}$ false	3.476109	0.07782

## Data Availability

The two real datasets were reported by [20].
